# Supplementary material for: Post-transcriptional regulation of aromatic amino acid metabolism by GcvB small RNA in Escherichia coli
Source: Microbiol Spectr. 2025 Jan 27;13(3):e02035-24. doi: 10.1128/spectrum.02035-24 (PMC11878033; doi:10.1128/spectrum.02035-24)
Supplement: Supplemental material — Tables S1 to S4. [file spectrum.02035-24-s0001.pdf]

Supplementary Table 1. Plasmids used in this study.

Name	Relevant fragment	Comment	Origin / marker	Reference
pTP11	Control plasmid	Control plasmid based on pJV300, ColE1 origin replaced by p15A origin	p15A / Amp^R	(11)
pP_L-*gcvB*	$P_{Llac\text{-}O}$ *gcvB*	*E. coli gcvB* mid-copy expression plasmid, *gcvB* is controlled by the constitutive P_{LlacO} promoter	p15A / Amp^R	(24)
pP_L-*gcvB*ΔR1	$P_{Llac\text{-}O}$-*gcvB* ΔR1	*E. coli gcvB* deletion of position 66 – 91	p15A / Amp^R	(24)
pP_L-*gcvB*ΔR13	$P_{Llac\text{-}O}$-*gcvB* ΔR1 ΔR3	*E. coli gcvB* deletion of position 66 – 91 and 152 – 169	p15A / Amp^R	(24)
pP_L-*gcvB*ΔR1mutR3	$P_{Llac\text{-}O}$-*gcvB* ΔR1, mutR3	*E. coli gcvB* ΔR1 mutant in position 154 – 158 (CTGTC->GACAG)	p15A / Amp^R	(24)
pP_L-*gcvB*ΔR1G156C	$P_{L\ lac\text{-}O}$-*gcvB* ΔR1, G156C	*E. coli gcvB* ΔR1 mutant in position 156 (G->C)	p15A / Amp^R	(24)
pP_L-*gcvB*ΔR1G160C	$P_{L\ lac\text{-}O}$-*gcvB* ΔR1, G160C	*E. coli gcvB* ΔR1 mutant in position 160 (G->C)	p15A / Amp^R	(24)
pP_L-*gcvB*ΔR1C162G	$P_{L\ lac\text{-}O}$-*gcvB* ΔR1, C162G	*E. coli gcvB* ΔR1 mutant in position 162 (C->G)	p15A / Amp^R	(24)
pKP8-35	Control plasmid	pBAD control plasmid, expresses ~50 nt nonsense RNA derived from *rrnB* terminator	pBR322 / Amp^R	(64)
pBAD-*gcvB*	P_{BAD}-*gcvB*	*E. coli gcvB* mid-copy expression plasmid, *gcvB* is controlled by the L-arabinose-inducible P_{BAD} promoter	pBR322 / Amp^R	This study
pXG-10sf	P_{LtetO}- *lacZ*::*gfp*	Plasmid for construction of translational sfGFP fusion	pSC101* / Cm^R	(26)
pXG-30sf	P_{LtetO}-FLAG::*glmU-glmS*::*gfp*	Plasmid for construction of translational sfGFP fusions of dicistronic targets	pSC101* / Cm^R	(26)
pXG-10sf-*aroG*	P_{LtetO}-*aroG*::*gfp*	*E. coli aroG* translational GFP fusion plasmid	pSC101*/ Cm^R	this study
pXG-10sf-*aroG*$_{G\text{-}8C}$	P_{LtetO}-*aroG*$_{G\text{-}8C}$::*gfp*	*E. coli aroG* mutant in position -8 relative to the start codon (G->C)	pSC101*/ Cm^R	this study
pXG-10sf-*aroF*	P_{LtetO}-*aroF*::*gfp*	*E. coli aroF* translational GFP fusion plasmid	pSC101*/ Cm^R	this study
pXG-10sf-*aroH*	P_{LtetO}-*aroH*::*gfp*	*E. coli aroH* translational GFP fusion plasmid	pSC101*/ Cm^R	this study
pXG-10sf-*aroK*	P_{LtetO}-*aroK*::*gfp*	*E. coli aroK* translational GFP fusion plasmid	pSC101*/ Cm^R	this study
pXG-10sf-*aroL*	P_{LtetO}-*aroL*::*gfp*	*E. coli aroL* translational GFP fusion plasmid	pSC101*/ Cm^R	this study
pXG-10sf-*serC*	P_{LtetO}-*serC*::*gfp*	*E. coli serC* translational GFP fusion plasmid	pSC101*/ Cm^R	this study
pXG-10sf-*aroA*	P_{LtetO}-*aroA*::*gfp*	*E. coli aroA* translational GFP fusion plasmid	pSC101*/ Cm^R	this study
pXG-10sf-*pheA*	P_{LtetO}-*pheA*::*gfp*	*E. coli pheA* translational GFP fusion plasmid	pSC101*/ Cm^R	this study
pXG-10sf-*tyrB*	P_{LtetO}-*tyrB*::*gfp*	*E. coli tyrB* translational GFP fusion plasmid	pSC101*/ Cm^R	this study
pXG-10sf-*pheP*	P_{LtetO}-*pheP*::*gfp*	*E. coli pheP* translational GFP fusion plasmid	pSC101*/ Cm^R	this study
pXG-10sf-*tyrP*	P_{LtetO}-*tyrP*::*gfp*	*E. coli tyrP* translational GFP fusion plasmid	pSC101*/ Cm^R	this study
pXG-10sf-*mtr*	P_{LtetO}-*mtr*::*gfp*	*E. coli mtr* translational GFP fusion plasmid	pSC101*/ Cm^R	this study
pXG-10sf-*tnaC*	P_{LtetO}-*tnaC*::*gfp*	*E. coli tnaC* translational GFP fusion plasmid	pSC101*/ Cm^R	this study
pXG-10sf-*tnaA*	P_{LtetO}-*tnaA*::*gfp*	*E. coli tnaA* translational GFP fusion plasmid	pSC101*/ Cm^R	this study
pXG-30sf-*aroK-aroB*	P_{LtetO}-FLAG::*aroK-aroB*::*gfp*	*E. coli aroK-aroB* translational GFP fusion plasmid	pSC101*/ Cm^R	this study
pXG-30sf-*ydiB-aroD*	P_{LtetO}-FLAG::*ydiB-aroD*::*gfp*	*E. coli ydiB-aroD* translational GFP fusion plasmid	pSC101*/ Cm^R	this study

pXG-30sf-*tsaC-aroE*	P_{LtetO}-FLAG::*tsaC-aroE*::*gfp*	*E. coli tsaC-aroE* translational GFP fusion plasmid	pSC101*/ Cm^R	this study
pXG-30sf-*trpE-trpD*	P_{LtetO}-FLAG::*trpE-trpD*::*gfp*	*E. coli trpE-trpD* translational GFP fusion plasmid	pSC101*/ Cm^R	this study
pXG-30sf-*trpD-trpC*	P_{LtetO}-FLAG::*trpD-trpC*::*gfp*	*E. coli trpD-trpC* translational GFP fusion plasmid	pSC101*/ Cm^R	this study
pXG-30sf-*trpC-trpB*	P_{LtetO}-FLAG::*trpC-trpB*::*gfp*	*E. coli trpC-trpB* translational GFP fusion plasmid	pSC101*/ Cm^R	this study
pXG-30sf-*trpB-trpA*	P_{LtetO}-FLAG::*trpB-trpA*::*gfp*	*E. coli trpB-trpA* translational GFP fusion plasmid	pSC101*/ Cm^R	this study
pXG-30sf-*aroF-tyrA*	P_{LtetO}-FLAG::*aroF-tyrA*::*gfp*	*E. coli aroF-tyrA* translational GFP fusion plasmid	pSC101*/ Cm^R	this study
pXG-30sf-*tnaA-tnaB*	P_{LtetO}-FLAG::*tnaA-tnaB*::*gfp*	*E. coli tnaA-tnaB* translational GFP fusion plasmid	pSC101*/ Cm^R	this study

Supplementary Table 2. DNA Oligonucleotides used in this study.

Sequences are given in 5´->3´ direction.

Name	Sequence	Used for
**Northern blot**		
MMO-1062	CTTTAAACATCTCCCTGAACCGTTC	Generation of *tnaA* antisense probe
MMO-1704	GTTTTTTTTAATACGACTCACTATAGGGTGCCGCTGTCGGTCAGTAAATCG	
JVO-0750	AATCACTATGGACAGACAGGGTA	GcvB oligo probe
MMO-1056	ACTACCATCGGCGCTACGGC	5S rRNA oligo probe
**GcvB cloning**		
JVO-0895	ACTTCCTGAGCCGGAAC	GcvB cloning into the pBAD plasmid
MMO-0086	GTTTTTTCTAGATAACGATACCGGTATGATTTC	
**GcvB target cloning**		
MMO-1363	GTTTTTATGCATTATTGCATTCACTAAGATAAGTATG	*aroG* GFP fusion cloning
MMO-1364	GTTTTTGCTAGCTTCAGTAGCGGGGAATTT	
MMO-1359	GTTTTTATGCATATCCTCGCTGAGGATCAAC	*aroF* GFP fusion cloning
MMO-1360	GTTTTTGCTAGCGCTCAATGGAAAAGCGGC	
MMO-1446	GTTTTTATGCATATCACTTCCCGGCAGTCCTG	*aroH* GFP fusion cloning
MMO-1447	GTTTTTGCTAGCACGGAGTTCGTCAGTTCTGTTCATTAC	
MMO-1451	GTTTTTATGCATGAAGAGATTGCCGACGTGACC	*aroK-aroB* GFP fusion cloning
MMO-1452	GTTTTTGCTAGCCCCGAGAGTAACGACAATCCTCTC	
MMO-1453	GTTTTTATGCATCAAGGGGCTGAACAGTTCAC	*ydiB-aroD* GFP fusion cloning
MMO-1455	GTTTTTGCTAGCACCAATGACGAGATCTTTTACAGTTAC	
MMO-1456	GTTTTTATGCATGTTGTGCCTGGTGAAACGGG	*tsaC-aroE* GFP fusion cloning
MMO-1457	GTTTTTGCTAGCGCTGTGGGCTATCGGATTACC	
MMO-1448	GTTTTTATGCATTCTGACTCTCGCAATATCTTATGAGG	*aroK* GFP fusion cloning
MMO-1449	GTTTTTGCTAGCCCCAACCAGAAAGATATTGCG	
MMO-1459	GTTTTTATGCATCGCATTGCGACCTATTGGGG	*aroL* GFP fusion cloning
MMO-1460	GTTTTTGCTAGCGACCGTTGTTTTACCACAGC	
MMO-2156	GTTTTTATGCATACCTGTGGTCGCAATCGA	*serC* GFP fusion cloning
MMO-2157	GTTTTTGCTAGCTTTAAGCACCTCTGCCGG	
MMO-1461	GTTTTTATGCATAGAGAGTTGAGTTCATGGAATCCC	*aroA* GFP fusion cloning
MMO-1462	GTTTTTGCTAGCGACACGAGCGATGGGTTGTAAC	
MMO-1426	GTTTTTATGCATCCGCAGTCGGAAGCCGAC	*trpD* GFP fusion cloning
MMO-1427	GTTTTTGCTAGCAATCACCACGTTATGCCCATTGC	
MMO-1424	GTTTTTATGCATGATCTGCAAGCCAATGCGC	*trpC* GFP fusion cloning
MMO-1425	GTTTTTGCTAGCCGTGCTCGGCTGAACCTCATTC	
MMO-1422	GTTTTTATGCATCTTGATTTTAATTCTGCTGTAGAGTCG	*trpB* GFP fusion cloning
MMO-1423	GTTTTTGCTAGCTTCTTCCAGCTGGCGCAGAG	
MMO-1420	GTTTTTATGCATGAGCAGCTACTGGTGGTTAAC	*trpA* GFP fusion cloning
MMO-2193	GTTTTTGCTAGCCTGGGCAAACAGAGATTCGTAG	
MMO-1357	GTTTTTATGCATATTGATAACAAAAAGGCAACACT	*pheA* GFP fusion cloning
MMO-1358	GTTTTTGCTAGCCAGTTTGGCTTTTCCCACC	
MMO-1361	GTTTTTATGCATGATGCCTGCATTAGCTGG	*aroF-tyrA* GFP fusion cloning
MMO-1362	GTTTTTGCTAGCAACCAGTTCCAGACGCTT	

MMO-1463	GTTTTTATGCATACCACCTGCCCGTAAACCTG	*tyrB* GFP fusion cloning
MMO-1464	GTTTTTGCTAGCAATTCCGTCTTCGTTGTAGTACAGAC	
MMO-0895	GTTTTTATGCATCTCAACAAAAAAGACACACAGGG	*pheP* GFP fusion cloning
MMO-0896	GTTTTTGCTAGCACCAGTACCAATTGCGCC	
MMO-1978	GTTTTTATGCATGTGTCTTGCGAGGATAAGTGC	*tnaC* GFP fusion cloning
MMO-1979	GTTTTTGCTAGCAGGGCGGTGATCGACAATTTTG	
MMO-1983	GTTTTTGCTAGCCATTACATAATCCTTCATTTATTTTAATTACAG	*tnaA* GFP fusion cloning
MMO-0934	GTTTTTATGCATAAAGAGAACGCGGCGAATA	*tnaA-tnaB* GFP fusion cloning
MMO-0935	GTTTTTGCTAGCACCTGCTATAACCATAACACCC	
MMO-0927	GTTTTTATGCATATATCACTCATAAAGATCGT	*tyrP* GFP fusion cloning
MMO-0928	GTTTTTGCTAGCCAACAAGATTAACGTAACG	
MMO-0932	GTTTTTATGCATATAACAACGCAGTCGCACT	*mtr* GFP fusion cloning
MMO-0933	GTTTTTGCTAGCGATAATCACCACGCCGCC	
**GcvB target mutagenesis**		
MMO-1395	ACACTGCAACAGACATGAATTATCAG	*aroG* G-8C
MMO-1396	TCTGTTGCAGTGTTGCCATACTTATC	
**qRT-PCR**		
MMO-1478	AGTCAGGGATGCTGAAGCTTGG	Quantification of *dppA* mRNA levels
MMO-1479	TGCGGGTTAAACCCTTCCGG	
MMO-1696	ACCGTATGTTAAACCGGGCG	Quantification of *map* mRNA levels
MMO-1697	GATGCAAACGGATTTCGGATAGC	
MMO-1480	AGAGTTACTTCCTCCTGTCGC	Quantification of *aroG* mRNA levels
MMO-1481	GGATCTTATGGATCGCTTTTCGG	
MMO-2103	ACGTACATATTACCGACGAACAGG	Quantification of *aroF* mRNA levels
MMO-2104	GATAATATCTGAAATGCTTTTACGCGAG	
MMO-1570	ACAGAACTGACGAACTCCGTAC	Quantification of *aroH* mRNA levels
MMO-1571	CAACAGTCGCTTATCTTCACCATTC	
MMO-1550	AGGATTGTCGTTACTCTCGGG	Quantification of *aroB* mRNA levels
MMO-1551	CGATTTCAGCGGTAAGAATGAAGC	
MMO-2105	ATCTCGTCATTGGTACGGGC	Quantification of *aroD* mRNA levels
MMO-2106	TTCACGATAGGCGAGAGCTTC	
MMO-1552	GGTAATCCGATAGCCCACAGC	Quantification of *aroE* mRNA levels
MMO-1553	CATTGATGGGTGCCAACACG	
MMO-2107	GCAATATCTTTCTGGTTGGGCC	Quantification of *aroK* mRNA levels
MMO-2108	GTTCGTTTCTCAATCTCTTGATCGG	
MMO-1554	GCTGTGGTAAAACAACGGTCG	Quantification of *aroL* mRNA levels
MMO-1555	GAGCTGTGATTGCAACCACTG	
MMO-2191	CTTCAATTTTAGTTCTGGTCCG	Quantification of *serC* mRNA levels
MMO-2192	ACTTCCATCACCGACGTAC	
MMO-2109	CGTGTCGATGGCACTATTAATCTG	Quantification of *aroA* mRNA levels
MMO-2110	AGATTGGTTAATACTGTTTTGCCG	
MMO-1672	TTTCGCGTAACCACCTTCGG	Quantification of *aroC* mRNA levels
MMO-1673	GGGTGGTATAGCGCGATGTC	
MMO-1674	AACTGCTAACCTGCGAAGGC	Quantification of *trpE* mRNA levels

MMO-1675	GCTGTCGATATCTGCGGATTCC	
MMO-1676	GATCAGTTGCGCAGCAATGG	Quantification of *trpD* mRNA levels
MMO-1677	GGCCAGGAGAAAGCATCAGC	
MMO-1560	GAAAATCGTCGCAGACAAGGC	Quantification of *trpC* mRNA levels
MMO-1561	CGCTTTCTTGCACTCCAGAATAAAC	
MMO-1678	CATGTACGTGCCACAAATCCTG	Quantification of *trpB* mRNA levels
MMO-1679	GACGCCCGGCATAGTTTTTC	
MMO-1562	CGAATCTCTGTTTGCCCAGTTG	Quantification of *trpA* mRNA levels
MMO-1563	TTTTCAATGACTGCTCAATGCCC	
MMO-1482	GCTGCGAGAGAAAATCAGCG	Quantification of *pheA* mRNA levels
MMO-1483	GCGATGCGAGAGCAGTTTGG	
MMO-1556	TTGCTGAATTGACCGCATTACG	Quantification of *tyrA* mRNA levels
MMO-1557	CCACTTCAGCAACCAGTTCC	
MMO-1558	ACCCGATTCTTACGCTTATGGAG	Quantification of *tyrB* mRNA levels
MMO-1559	CAGTTGTGGAATAATTCCGTCTTCG	
MMO-1670	ATTCAGCTTATCGCGCTGGG	Quantification of *aroP* mRNA levels
MMO-1671	GGCGATAAAACCAGCAATGGC	
MMO-1564	TCAACCGTATCGGAAGATACTGC	Quantification of *pheP* mRNA levels
MMO-1565	AGACCAGTACCAATTGCGCC	
MMO-1568	GCTATTACTGCTGGAGGTGTACC	Quantification of *tyrP* mRNA levels
MMO-1569	CTGATGTATGCCGCAGTCAGAG	
MMO-1566	ACTAACCACCACCCAAACGTC	Quantification of *mtr* mRNA levels
MMO-1567	TAAAGATCAGCGCCGCCATTG	
MMO-1139	TTGCGAGGATAAGTGCATTATGAATATC	Quantification of *tnaC* mRNA levels
MMO-1140	TCAAGGGCGGTGATCGACAATTTTG	
MMO-1062	CTTTAAACATCTCCCTGAACCGTTC	Quantification of *tnaA* mRNA levels
MMO-1063	GTGCCGCTGTCGGTCAGTAAATCG	
MMO-1197	GGGTGCCTTTATCCTTATCATTGCC	Quantification of *tnaB* mRNA levels
MMO-1198	GCTGATAATGTTCCAGGTGTTACCG	

Supplementary Table 3. Details of GFP fusion plasmids.

Target gene	Backbone	Oligos used to amplify insert	Insert digested with	Upstream ORF [bp]	Intergenic region [bp]	Downstream ORF [bp]	Insert length [bp]	Translational fusion to N-terminal FLAG [aa]	Translational fusion to C-terminal GFP [aa]
aroG	pXG-10sf	MMO-1363 x MMO-1364	NsiI/NheI	-	42	90	132	-	30
aroF	pXG-10sf	MMO-1359 x MMO-1360	NsiI/NheI	-	51	90	141	-	30
aroH	pXG-10sf	MMO-1446 x MMO-1447	NsiI/NheI	-	51	24	75	-	8
aroB	pXG-30sf	MMO-1451 x MMO-1452	NsiI/NheI	90	56	27	173	30	9
aroD	pXG-30sf	MMO-1453 x MMO-1455	NsiI/NheI	84	30	36	150	28	12
aroE	pXG-30sf	MMO-1455 x MMO-1456	NsiI/NheI	84	4	42	130	28	14
aroK	pXG-10sf	MMO-1448 x MMO-1449	NsiI/NheI	-	103	33	136	-	11
aroL	pXG-10sf	MMO-1459 x MMO-1460	NsiI/NheI	-	30	54	84	-	18
serC	pXG-10sf	MMO-2156 x MMO-2157	NsiI/NheI	-	58	60	118	-	20
aroA	pXG-10sf	MMO-1461 x MMO-1462	NsiI/NheI	-	14	36	50	-	12
trpD	pXG-30sf	MMO-1426 x MMO-1427	NsiI/NheI	90	-1	90	179	30	30
trpC	pXG-30sf	MMO-1424 x MMO-1425	NsiI/NheI	90	0	111	201	30	37
trpB	pXG-30sf	MMO-1422 x MMO-1423	NsiI/NheI	90	11	90	191	30	30
trpA	pXG-30sf	MMO-1420 x MMO-2193	NsiI/NheI	90	-1	30	119	30	10
pheA	pXG-10sf	MMO-1357 x MMO-1358	NsiI/NheI	-	23	120	143	-	40
tyrA	pXG-30sf	MMO-1361 x MMO-1362	NsiI/NheI	90	10	90	190	30	30
tyrB	pXG-10sf	MMO-1463 x MMO-1464	NsiI/NheI	-	32	126	158	-	42
pheP	pXG-10sf	MMO-0895 x MMO-0896	NsiI/NheI	-	30	120	150	-	40
tnaC	pXG-10sf	MMO-1978 x MMO-1979	NsiI/NheI	-	24	72	96	-	24
tnaA	pXG-10sf	MMO-1978 x MMO-1983	NsiI/NheI	-	319	3	322	-	1
tnaB	pXG-30sf	MMO-0934 x MMO-0935	NsiI/NheI	90	90	60	240	30	20
tyrP	pXG-30sf	MMO-0927 x MMO-0928	NsiI/NheI	-	36	120	156	-	40
mtr	pXG-30sf	MMO-0932 x MMO-0933	NsiI/NheI	-	43	60	103	-	20

Supplementary Table 4. Inserts of GFP fusion plasmids.

GFP fusion	Insert
aroG	ATGCATtattgcattcactaagataagtatggcaacactggaacagacatgaattatcagaacgacgatttacgcatcaaagaaatcaaagagttacttcctcctgtcgcattgctggaaaaattccccgctactgaaGCTAGC
aroF	ATGCATatcctcgctgaggatcaactatcgcaaacgagcataaacaggatcgccatcatgcaaaaagacgcgctgaataacgtacatattaccgacgaacaggttttaatgactccggaacaactgaaggccgcttttccattgagcGCTAGC
aroH	ATGCATatcacttcccggcagtcctgccgtagaagcaacaaatttctgagacttgtaatgaacagaactgacgaactccgtGCTAGC
aroK-aroB	ATGCATgaagagattgccgacgtgaccattcgtactgatgatcaaagcgctaaagtggttgcaaaccagattattcacatgctggaaagcaactaattctggctttatatacactcgtctgcgggtacagtaattaaggtggatgtcgcgttatggagaggattgtcgttactctcgggGCTAGC
ydiB-aroD	ATGCATcaaggggctgaacagttcacattatggactggcaaagatttccctctggaatatgttaaacaggtcatggggttcggtgcctgacaggctgaccgcgtgcagaaagggtaaaaaatgaaaaccgtaactgtaaaagatctcgtcattggtGCTAGC
tsaC-aroE	ATGCATgttgtgcctggtgaaacgggggggcgtttaaatccttcagaaatccgcgatgccctgacgggtgaactgtttcgacaggggtaacataatggaaacctatgctgtttttggtaatccgatagcccacagcGCTAGC
aroK	ATGCATtctgactctcgcaatatcttatgaggtttcagttcatgtcctgcggcgctctctgagcgaagcgggtttatcattaacgaatagtcttagtagtaccgaaaaaatggcagagaaacgcaatatctttctggttgggGCTAGC
aroL	ATGCATcgcattgcgacctattggggaaaacccacgatgacacaacctctttttctgatcgggcctcggggctgtggtaaaacaacggtcGCTAGC
serC	ATGCATacctgtggtcgcaatcgattgaccgcgggttaatagcaacgcaacgtggtgaggggaaatggctcaaatcttcaattttagttctggtccggcaatgctaccggcagaggtgcttaaaGCTAGC
aroA	ATGCATagagagttgagttcatggaatccctgacgttacaacccatcgctcgtgtcGCTAGC
trpE-trpD	ATGCATccgcagtcggaagccgacgaaacccgtaacaaagcccgcgctgtactgcgcgctattgccaccgcgcatcatgcacaggagactttctgatggctgacattctgctgctcgataatatcgactcttttacgtacaacctggcagatcagttgcgcagcaatgggcataacgtggtgattGCTAGC
trpD-trpC	ATGCATgatctgcaagccaatgcgcaaaccgttcttgaggtactgcgcagtggttccgcttacgacagagtcaccgcactggcggcacgagggtaaatgatgcaaaccgttttagcgaaaatcgtcgcagacaaggcgatttgggtagaagcccgcaaacagcagcaaccgctggccagttttcagaatgaggttcagccgagcacgGCTAGC
trpC-trpB	ATGCATcttgattttaattctgctgtagagtcgcaaccgggcatcaaagacgcacgtcttttggcctcggttttccagacgctgcgcgcatattaaggaaaggaacaatgacaacattacttaacccctattttggtgagtttggcggcatgtacgtgccacaaatcctgatgcctgctctgcgccagctggaagaaGCTAGC
trpB-trpA	ATGCATgagcagctactggtggttaacctttccggtcgcggcgataaagacatcttcaccgttcacgatattttgaaagcacgaggggaaatctgatgggaacgctacgaatctctgtttgcccagGCTAGC
pheA	ATGCATattgataacaaaaaggcaacactatgacatcggaaaacccgttactggcgctgcgagagaaaatcagcgcgctggatgaaaaattattagcgttactggcagaacggcgcgaactggccgtcgaggtgggaaaagccaaactgGCTAGC
aroF-tyrA	ATGCATgatgcctgcattagctgggaaatgaccgatgccttgctgcgtgaaattcatcaggatctgaacgggcagctgacggctcgcgtggcttaagaggtttattatggttgctgaattgaccgcattacgcgatcaaattgatgaagtcgataaagcgctgctgaatttattagcgaagcgtctggaactggttGCTAGC
tyrB	ATGCATaccacctgcccgtaaacctggagaaccatcgcgtgtttcaaaaagttgacgcctacgctggcgacccgattcttacgcttatggagcgttttaaagaagaccctcgcagcgacaaagtgaatttaagtatcggtctgtactacaacgaagacggaattGCTAGC
pheP	ATGCATctcaacaaaaaagacacacaggggaaaggcgtgaaaaacgcgtcaaccgtatcggaagatactgcgtcgaatcaagagccgacgcttcatcgcggattacataaccgtcatattcaactgattgcgttgggtggcgcaattggtactggtGCTAGC
tnaC	ATGCATgtgtcttgcgaggataagtgcattatgaatatcttacatatatgtgtgacctcaaaatggttcaatattgacaacaaaattgtcgatcaccgccctGCTAGC
tnaA	ATGCATgtgtcttgcgaggataagtgcattatgaatatcttacatatatgtgtgacctcaaaatggttcaatattgacaacaaaattgtcgatcaccgcccttgatttgcccttctgtagccatcaccagagccaaaccgattagattcaatgtgatctatttgtttgctatatcttaattttgccttttgcaaaggtcatctctcgtttatttacttgttttagtaaatgatggtgcttgcatatatatctggcgaattaatcggtatagcagatgtaatattcacagggatcactgtaattaaaataaatgaaggattatgtaatgGCTAGC
tnaA-tnaB	ATGCATaaagagaacgcggcgaatattaaaggattaacctttacgtacgaaccgaaagtattgcgtcacttcaccgcaaaacttaaagaagtttaattaatactacagagtggctataaggatgttagccactctcttaccctacatcctcaataacaaaaatagccttcctctaaaggtggcatcatgactgatcaagctgaaaaaaagcactctgcattttggggtgttatggttatagcaggtGCTAGC
tyrP	ATGCATatatcactcataaagatcgtcaggacagaagaaagcgtgaaaaacagaaccctgggaagtgttttatcgtggcgggaaccacaattggcgcaggcatgctggcaatgccgctggctgcggccggtgttggttttagcgttacgttaatcttgttgGCTAGC
mtr	ATGCATataacaacgcagtcgcactattttcactggagagaagccctcatggcaacactaaccaccacccaaacgtcaccgtcgctgcttggcggcgtggtgattatcGCTAGC

For pXG-10sf derivatives, 5´UTR sequences are indicated in black and coding sequences are indicated in red. For the intraoperonic fusions in pXG30-sf, upstream ORF fused with FLAG, intergenic region, downstream ORF fused with GFP are indicated in blue, black, and red, respectively. The overlapping nucleotide between stop and start codons is highlighted in magenta. NsiI and NheI sites used for cloning are highlighted in bold in cyan and green, respectively.
